# Nanopore device-based fingerprinting of RNA oligos and microRNAs enhanced with an Osmium tag

**DOI:** 10.1038/s41598-019-50459-8

**Published:** 2019-10-02

**Authors:** Madiha Sultan, Anastassia Kanavarioti

**Affiliations:** Yenos Analytical LLC, 4659 Golden Foothill Pkwy, Suite 101, El Dorado Hills, CA 95672 USA

**Keywords:** RNAi therapy, Nanopores

## Abstract

Protein and solid-state nanopores are used for DNA/RNA sequencing as well as for single molecule analysis. We proposed that selective labeling/tagging may improve base-to-base resolution of nucleic acids via nanopores. We have explored one specific tag, the Osmium tetroxide 2,2′-bipyridine (OsBp), which conjugates to pyrimidines and leaves purines intact. Earlier reports using OsBp-tagged oligodeoxyribonucleotides demonstrated proof-of-principle during unassisted voltage-driven translocation via either alpha-Hemolysin or a solid-state nanopore. Here we extend this work to RNA oligos and a third nanopore by employing the MinION, a commercially available device from Oxford Nanopore Technologies (ONT). Conductance measurements demonstrate that the MinION visibly discriminates oligoriboadenylates with sequence A_15_PyA_15_, where Py is an OsBp-tagged pyrimidine. Such resolution rivals traditional chromatography, suggesting that nanopore devices could be exploited for the characterization of RNA oligos and microRNAs enhanced by selective labeling. The data also reveal marked discrimination between a single pyrimidine and two consecutive pyrimidines in OsBp-tagged A_n_PyA_n_ and A_n_PyPyA_n_. This observation leads to the conjecture that the MinION/OsBp platform senses a 2-nucleotide sequence, in contrast to the reported 5-nucleotide sequence with native nucleic acids. Such improvement in sensing, enabled by the presence of OsBp, may enhance base-calling accuracy in enzyme-assisted DNA/RNA sequencing.

## Introduction

Nanopores made critical strides the last 30 years due to the efforts of scientists and technologists who spearheaded nanopore-based DNA sequencing^1–11^. Specifically a protein nanopore, with sub 2 nm diameter, is inserted in a planar lipid bilayer membrane that separates two electrolyte filled compartments. Applying a voltage across the two compartments leads to a constant flow of electrolyte ions via the nanopore (*i-t*). This flow is reduced by the passage of a single molecule through the pore. In one approach, alpha-Hemolysin (α-HL) conjugated to a polymerase enzyme at the entry of the pore, directs the synthesis of the complementary strand of a target DNA. Upon elongation with polymer-conjugated mononucleotides, the cleaved polymer goes through and is detected by the pore^11,12^. Using a unique polymer tag for each nucleobase leads to sequencing-by-synthesis (SBS)^6,11,12^. A second approach, using α-HL^13–16^, Mycobacterium smegmatis porin A (MspA)^17–19^, or the proprietary nanopore of the MinION^20^, achieves direct sequencing of the target strand. Here a single stranded (ss) DNA/RNA strand moves through the pore assisted by an enzyme that acts as a motor to move the strand one nucleobase at a time slowing down the translocation to a rate amenable to measurement^21–23^.

In addition to sequencing, several nanopore platforms are successfully employed for nanoparticle or single molecule analyses^8^. Solid-state nanopores were used to fingerprint nucleic acid nanoparticles^24^, and for protein detection and quantitation^25^. α-HL was successfully used for polymer sizing^26^, for metallic nanoparticle characterization^27^, and to measure the kinetics of enzymatic phosphodiester bond formation^1,28^. A modified MspA was shown to measure the movement of enzymes along DNA in real time^29^, wt aerolysin to resolve a series of deoxyriboadenylates^30^, and an engineered Fragaceatoxin C to discriminate among peptides^31^. Cytolysin A nanopore was used to report the concentration of glucose and asparagine from samples of blood, and other bodily fluids^32^, introducing nanopores to the arena of non-invasive diagnostic assays.

Using a nanopore experimental platform is a complex process. Specifically, the monomeric unit of the nanopore protein needs to be manufactured, purified, assembled as a pore, and inserted in a premade stable lipid bilayer. This is followed by addition of the nucleic acid in a small reaction vessel without disrupting the lipid bilayer/nanopore construct^5^. In 2014 ONT commercialized a hand-held, relatively inexpensive device, the MinION. This device is equipped with an array consisting of over 2000 preassembled nanopores from a bioengineered pore-forming lipoprotein, CsgG^33^, and achieves DNA/RNA sequencing in conjunction with a laptop and the internet. The software of the device, MinKNOW, selects 512 nanopores with optimal properties, and records simultaneously 512 *i-t* traces that decode to nucleic acid sequence. Of these, a typical run initially has less than 500 pores and this number decreases over time as the pores degrade or become blocked. The *i-t* traces are also reported as raw data in *fast-5* file format, and can be visualized using MatLab from Mathworks. The availability and convenience of this device led us to evaluate it as a simple analytical tool for RNA oligos, and to compare it to traditional analytical tools such as high-performance liquid-chromatography (HPLC).

Voltage-driven translocation of RNA via a nanopore is very fast and measures 5 to 10 μs per base^1,2,5^. The MinION exhibits a 3.012 kHz sampling rate, equivalent to reporting 3 data points per 1 ms. This slow acquisition rate implies that DNA/RNAs, let us say 100 nucleotides (nt) or shorter, may not be detected/recorded by this device. In addition to our own experiments (see below), multiple reports confirm this expectation. In one study, attempts to sequence RNAs shorter than 200 nt following ONT protocol were unsuccessful^34^. In another study 100 nt deoxyoligos were successfully sequenced by the MinION, albeit only after circularization and rolling circle amplification^35^. Several experimental nanopore platforms have been successfully exploited for detection of short RNAs by using a target probe complementary to the desired RNA sequence^36–38^. Current technologies for “small RNA” identification/quantitation, such as microarray, sequencing (Ion Torrent or Illumina (small RNA-seq)), and qRT-PCR-based methods, are complex and have extensive resource demands^39,40^. All these different methods exploit base-pairing or SBS, and therefore misinterpret any post-transcriptionally modified bases^41^ that may be present in the target RNA. Short RNAs are found in biological fluids, are relatively stable, are likely to contain modified bases, and are shown to exhibit important regulatory functions (see discussion later). Hence it is important to find ways to properly characterize them, and establish the presence, identity, and abundance of any modified bases within.

To enable short RNA sequencing we proposed to leverage the physicochemical properties (differences) of the bases and selectively tag one or more with a bulky label^42,43^. Assuming the label has high specificity, selectivity, and exhibits negligible side-reactions, the bulkiness alone is expected to slow down translocation to measurable *i-t* levels. The presence of a bulky tag should lead to a markedly larger discrimination between tagged base and native base, compared to the discrimination between two native nucleobases. In turn, better discrimination may reduce *i-t* dependence from the reported 5 nt sequence^34^ to, perhaps, a single labeled base. The selectivity/reactivity of the label for one base over another –see below, for example the selectivity of OsBp for Thymine (T) over Cytosine (C) - might enable base identification for some among the known 150 post-transcriptionally modified bases^41^. Our approach in passing the tagged nucleic acid via a nanopore is different from the one described above (SBS), where only the tag(s) are passing via the nanopore^11,12^, which is intrinsically blind to post-transcriptional modifications.

The reactivity of OsBp, known to add to the C5-C6 double bond of the pyrimidines, was established in the 1970s^44–47^. Recent work showed that OsBp addition to DNA, coined here osmylation, using Yenos’ proprietary protocol is a remarkably clean reaction. Osmylation yields pyrimidine conjugates in practically 100% yield with no detectable reactivity towards purines, and no phosphodiester bond cleavage^42,43,48^. The selectivity of osmylation for deoxythymidine (dT) over deoxycytosine (dC), is 30-fold, and leads to labeling of either mostly dT, or both dT + dC using low or high OsBp concentration, respectively^42^. Using a training set of deoxyoligos a UV-Vis assay was developed and validated to measure the extent of osmylation^42^ (see later). Monitoring the osmylation kinetics by HPLC or capillary electrophoresis (CE) resolved all the intermediate products, and led to the conclusion that the process is kinetically independent of sequence, length, and composition. As shown with M13mp18, a circular 7249 nt ssDNA, secondary structure does not affect the efficiency of osmylation, most likely due to a denaturing effect exhibited by the OsBp reagent^43^. The same protocol yields practically 100% pyrimidine osmylation in both short and long ssDNA^43^.

The outstanding labeling features of OsBp led us to undertake nanopore-based single molecule translocation experiments in a number of collaborating laboratories both in industry and academia. Pore size suitability using solid-state silicon nitride (SiN) nanopores showed that 1.6 nm wide pores permit translocation of 80 nt long osmylated deoxyoligos, and demonstrated dramatic tranlocation slowdown with increasing osmylation^49^. Experiments with dA_10_dPydA_9_ via wt α-HL showed readably slow and distinct translocation features for different deoxypyrimidines (dPy)^50^. Specifically, when dPy = dA, dT(OsBp), dC(OsBp), dU(OsBp), or 5-MedC(OsBp) the distribution of the fractional residual ion current, *I*_*r*_*/I*_*o*_, where *I*_*o*_ is open pore ion current and *I*_*r*_ is the residual ion current of the blockade or event has a maximum at 0.14, 0.08, 0.11, 0.12 and 0.12 (with STD ± 0.01), respectively. Similarly the observed duration or dwell times (τ) measured at −120 mV are 50, 150, 310, 360 and 470μs, respectively^50^. The effects are small with respect to residual ion current differences, but substantial with respect to translocation duration for oligos with a single dPy(OsBp)^50^. The collective data in two distinct nanopore platforms illustrate a remarkable slow down and visible discrimination between intact and osmylated DNA oligos.

The above findings, namely that DNA oligos can be selectively tagged on the pyrimidines and that these tagged oligos exhibit pyrimidine-dependent translocation features, led us to extend the work to RNA oligos. In addition, we now make use of a commercially available nanopore-device, so that the experiments can be reproduced by other scientists less familiar with the nanopore field. RNA oligos in the range of 20 to 300 nt include coding and non-coding nucleic acids with known functions in mRNA translation and in regulation of most biological processes^51–53^. microRNA (miRNA) is a group of short RNAs, 17 to 25 nt long, with universal regulatory functions^51^ and with applications in personalized medicine as biomarkers and potential therapeutics^53–56^. Due to the interest in short RNA characterization we extend here our osmylation protocol to RNA. Here we report that due to the mildness and selectivity of our osmylation protocol OsBp exhibits excellent labeling properties with RNA pyrimidines, comparable to that described above for DNA pyrimidines. Using the MinION and high quality synthetic RNA oligos (see Table 1) we show that unlabeled 31 nt RNAs are not detected by this device, but that the corresponding osmylated oligos undergo voltage-driven translocation, and are easily resolved from the noise, with as few as a single OsBp moiety. We further show that the reported fractional residual level of ion current, *I*_*r*_*/I*_*o*_, can serve as a fingerprint for each RNA. To the best of our knowledge this study is the first to place side-by-side HPLC profiles of intact RNAs^57^ with nanopore-derived fingerprints of the corresponding RNA(OsBp). Within the stochastic nature of the translocation process, a small number of long translocations yielded a rudimentary sequence pattern in the form of pyrimidine-purine (Py-Pu), that may be sufficient for short RNA characterization in a mixture. These observations serve as proof-of-principle for using commercially available nanopore device(s) as auxiliary analytical tools for short RNAs and miRNAs enhanced with an Osmium tag.

**Table 1:** List of tested RNA oligos.

RNA oligo (ID)	Sequence	Py/nt	R(312/272)^a^	(*I*_*r*_*/I*_*o*)max_^b^	Fig.
T11(ACA)	A_15_**C**A_15_	1/31	0.063	0.08, 0.15, 0.19, 0.23	S1
T12(AUA)	A_15_**U**A_15_	1/31	0.076	0.12, 0.17	S2
T13(5-MeC)	A_15_**(5-Me)C**A_15_	1/31	0.057	0.03	S3
T14(GCG)	A_14_G**C**GA_14_	1/31	0.071	0.13, 0.21	S4
T15(GUG)	A_14_G**U**GA_14_	1/31	0.065	0.13	S5
T16(5-MeU)	A_15_**(5-Me)U**A_15_	1/31	0.055	0.03	S6
T17(4-SU)^c^	A_15_**(4-S)U**A_15_	1/31	0.09	0.27	S7
T31(CC)	A_10_**CC**A_10_	2/22	0.18	0.03	S8
T32(UU)	A_10_**UU**A_10_	2/22	0.18	0.06	S9
T3(UU-UU)	A_15_**UU**A_19_**UU**A_15_	4/53	0.16	0.03, 0.13	S10
T4(CC-UU)	A_15_**CC**A_19_**UU**A_15_	4/53	0.16	0.03, 0.11	S11
T6(UU-UU-UU)	A_15_**UU**A_19_**UU**A_19_**UU**A_15_	6/74	0.16	0.03, 0.13	S12
T7(13Py)	(AG)_3_**C**_**4**_(AG)_3_**C**_**4**_(AG)_3_**CCUUC**A	13/32	0.72	0.01	S13
T8(9Py)	(AG)_4_**C**_**2**_(AG)_4_**C**_**2**_(AG)_3_**CCUUC**A	9/32	0.50	0.01	S14
miRNA122-5p	**U**GGAG**U**G**U**GA**C**AA**U**GG**U**G**UUU**G	9/22	0.82	0.04	S15
miRNA140-5p	**C**AG**U**GG**UUUU**A**CCCU**A**U**GG**U**AG	12/22	1.09	0.04	S16
T1	Sequence^d^	46/100	0.92	ND	4e, 7c

RNA oligo ID, sequence (all ribonucleotides), number of pyrimidines over total nucleotides (Py/nt), observed Absorbance ratio of the osmylated derivative at 312 nm vs. 272 nm (R(312/272)), fractional residual ion current maxima ((*Ir/Io)max*) from the histogram plots (Fig. 5), and analytical data collection for the specific RNA (Fig. S#) in the Supplementary Information.

ND, stands for not determined.

^a^Observed R(312/272) from HPLC and/or CE. Practically 100% osmylation exhibits theoretical R(312/272) = 2xPy/nt, and equals 0.065, 0.18, 0.15, 0.16, 0.81, 0.56, 0.82, 1.1, and 0.92 for Py/nt 1/31, 2/22, 4/53, 6/74, 13/32, 9/32, 9/22, 12/22 and 46/100, respectively. With the exception of T17, all intact oligos tested here exhibit R(312/272) = 0.01.

^b^For data acquisition, see text.

^c^Intact T17 has R(312/272) = 0.05 due to the presence of C = S.

^d^T1 sequence: 5′-UUA CAG CCA CGU CUA CAG CAG UUU UAG AGC UAG AAA UAG CAA GUU AAA AUA AGG CUA GUC CGU UAU CAA CUU GAA AAA GUG GCA CCG AGU CGG UGC UUU U-3′.

## Results and Discussion

### Labeling RNA pyrimidines with an Osmium tag: Manufacturing protocol and Stability

**Figure 1:**
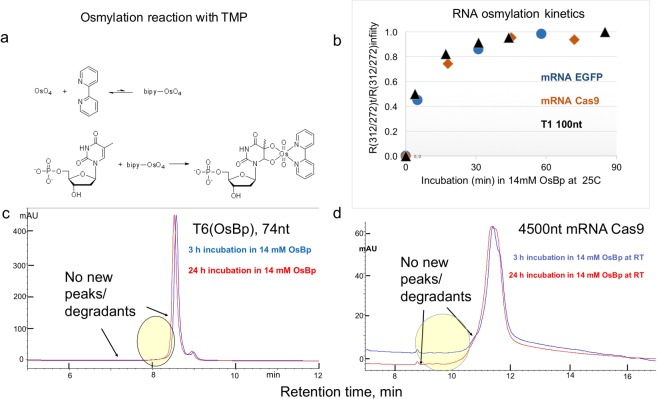
(**a**) Osmylation of Pyrimidines. Preequilibrium of Osmium tetroxide (OsO_4_) with 2,2′-bipyridine (bipy) to form a weak complex bipy-OsO_4_ or OsBp. In the following step OsBp adds to the C5-C6 double bond of a pyrimidine, thymidine monophosphate (TMP) shown here. Reaction of OsBp occurs from either side of the double bond and leads to topoisomers, often observed at 1:1 ratio. Due to the top/bottom addition the direction of the conjugate becomes parallel to the strand direction and the bulky OsBp extends all the way to the neighbor base. Evidence clearly shows that adjacent pyrimidines are kinetically as easily labeled as a monomer, as shown by dTTP being labeled as fast and as complete as oligo dT_15_^42^. Even though labeling is not slowed down within a long sequence of pyrimidines, translocation via nanopores is (see later). **(b)** RNA Osmylation Kinetics. Comparable kinetics (half-life of about 25 min) with 14 mM OsBp at 25 °C were observed with a 100 nt single guide RNA (sgRNA), a 1000 nt mRNA EGFP and a 4500 nt mRNA Cas9. Because the fraction of pyrimidines/ # of total nt is not equal for these three RNAs, observed absorbance ratio (R(312)/(272))_t_ at time t was normalized and plotted as a function of incubation time t. Normalization was done against the observed infinity value (R(312)/(272))_infinity_. **(c)** Stability-indicating HPLC profile at 260 nm of 74 nt T6 (Table 1) in 14 mM OsBp for 3 or 24 h. No new or increasing peaks were detected in the area in front of the main peak, consistent with undetectable degradation. Samples were quenched by removal of the excess label, and 3 h samples were kept at −20 °C until analysis, conducted at the same time as the 24 h samples. The T6 sample is rather concentrated, so that even degradants at 0.1% of T6 could be detected. IEX HPLC method at pH 12 (see Experimental Section). **(d**) Stability-indicating HPLC profile at 260 nm of 4,500 nt mRNA Cas9 in 14 mM OsBp for 3 or 24 h. Incubation, purification and HPLC analysis as described in (**c**) above.

Osmylation conditions for DNA were developed earlier^42,43^; these conditions were reevaluated, and optimized for RNAs. Briefly the RNA osmylation protocol requires 3 hour incubation in glass vials at room temperature in water with no buffer in the presence of 12 to 14 mM OsBp (1:1 equimolar mixture of OsO_4_ and 2,2′-bipyridine, Fig. 1a). The process yields practically 100% osmylation of the pyrimidines, even with long RNAs, like mRNA Cas9, known to exhibit secondary structure (Fig. 1b). It is important that the ratio of mM OsBp to mM pyrimidine, in monomer equivalents, is 30-fold or more, so that the labeling is not slowed down by depletion of OsBp, while it reacts with the oligo or evaporates (see Experimental Section). The high excess of OsBp reagent to pyrimidine monomer is also required due to the low association constant between OsO_4_ and 2,2′-bipyridine. The low association constant is evidenced by the square dependence of the osmylation kinetics on the reagent’s nominal concentration (Table S1 and Fig. S17 in the Supplementary Information).

Osmylation kinetics at the above manufacturing conditions are independent of RNA length, composition, and secondary structure, as seen in Fig. 1b. Specifically three RNAs of vastly different length (a 100 nt long single guide RNA (sgRNA), a 1000 nt long RNA, mRNA EGFP, and a 4500 nt long RNA, mRNA Cas9) conform onto the same kinetics with about 25 min half-life. The observation that nucleic acids with secondary structure, such as mRNAs, label kinetically equally fast as an oligo, is likely due to a denaturing effect of OsBp solutions at concentrations as high or higher than 12 mM^43^. In order to determine selectivity, the osmylation kinetics are conducted at a lower concentration of OsBp (3 to 6 mM). Osmylation kinetics were monitored automatically every 15 min using CE, and the CE profiles are included in the corresponding figure of the oligo in the Supplementary Information. With the exception of the oligoriboadenylate with 5-MeU substitution, which is subject to very fast osmylation, just as with the deoxy derivative^42^, all other tested oligos exhibit comparable reactivity. Observed relative reactivity towards osmylation obtained from the kinetics of T11 though T16 using 5.2 mM OsBp at 26 °C in water are U/C = 4.7, 5-MeC/U = 0.9, 5-MeC/C = 4.1 and 5-MeU/C = 44. This trend is in excellent agreement with the relative reactivity observed in deoxy oligos^50^. Osmylation reactivity depends on the electrophilicity of the C5-C6 double bond, and this is the feature that will enable selectivity of one vs. another nucleobase among some of the post-transcriptionally modified RNA bases^41^. Typically the reactivity of a base in a sequence mirrors the reactivity observed with the mononucleotide.

Removal of the excess OsBp after manufacturing takes about 7 minutes using a TrimGen mini-column following the manufacturer’s instructions. Extent of purification can be assessed by CE or HPLC (for methods see Experimental Section), because OsBp migrates (CE) or elutes (HPLC) well ahead of the oligo and of the osmylated product. However UV-Vis spectrophotometric measurements will not differentiate between label and labeled oligo, and therefore removal of the label is necessary. HPLC/CE profiles for each tested intact oligo and its fully osmylated derivative are included in the corresponding figure in the Supplementary Information. Osmylation does not lead to side-reactions, as can be seen in these profiles. In addition, the stability of the RNA in the presence of OsBp was evaluated with an extra pure 74 nt long RNA and with a 4500 nt long mRNA Cas9 using a stability-indicating HPLC method^57^ (see Experimental Section). Comparison between the HPLC profiles after 3 h and 24 h of osmylation (see Fig. 1c with 74 nt long RNA and Fig. 1d with mRNA Cas9) illustrates no detectable changes, suggesting no detectable degradation during an additional 21 h prolonged incubation.

Selective labeling of a nucleic acid requires an assay for quality control. It turns out that addition of OsBp to the C5-C6 Py double bond and formation of Py(OsBp) creates a new chromophore in the wavelength range of 300 to 320 nm, where nucleic acids exhibit negligible absorbance. We exploited this observation and used a deoxyoligo training set to show that extent of osmylation can be measured using the equation R(312/272) = 2 × (# of osmylated pyrimidines/total # of nucleotides). R(312/272) is the ratio of the observed absorbance at 312 nm over the observed absorbance at 272 nm^42,43^. Using the ratio R instead of the absorbance at 312 nm serves to normalize the measurement, and minimize instrument sampling variation. When experimental value R(312/272) is equal to 2 × (# of pyrimidines/total # of nucleotides), osmylation is practically 100% complete^42,43^. The wavelengths 312 nm and 272 nm were chosen in order to maximize the effect and to equalize contributions by osmylated dT, dC or dU, assuring that nucleic acid composition doesn’t affect the assay’s accuracy which stands at ±3%. The R(312/272) data obtained in this study (Table 1) confirm that the above equation is also valid for osmylated RNA with bases U, C, 5-MeU, and 5-MeC. The UV-Vis assay should be tested and confirmed or modified for other non-canonical bases.

### HPLC analysis of RNA oligos

**Figure 2:**
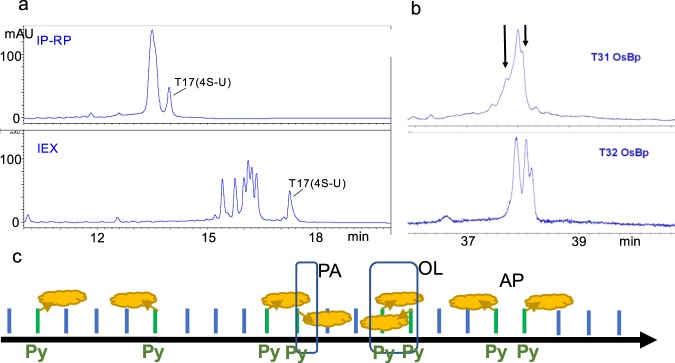
(**a**) HPLC profiles of an equimolar mixture of 31 nt intact RNAs T11 through T17. Oligos exhibit minimal sequence differences (see Table 1); they were analyzed by two HPLC methods, an ion-pair reversed phase (IP-RP) (top) and an ion-exchange (IEX) (bottom). Both profiles show that only T17(4-SU) elutes with baseline resolution from the others, and the other six RNAs elute closely together but resolve better via IEX compared to IP-RP. **(b**) CE profiles of the osmylated T31(CC) and T32(UU) show the presence of three diastereomers. The arrows in T31 indicate that the shoulders around the main peak are attributed to two isomers. For statistical reasons the theoretical ratio is PA:OL:AP = 2:1:1 (see cartoon in c). Observed ratio is close to the theoretical, only that PA configuration appears to migrate in the middle with T31 and first with T32. **(c)** Cartoon to show OsBp configurations on an RNA strand. RNA strand (black arrow), pyrimidines (Py, green bars), purines (Pu, blue bars), OsBp (yellow cloud conjugated to a Py by top or bottom addition). Left, two topooisomers for a single Py(OsBp) in a strand. Right, three possible topoisomers for a sequence with two consecutive Py(OsBp). In the first configuration the two OsBp moieties are lined up in the same direction (PA). In the middle configuration the two OsBp moieties are antiparallel, but lined towards each other (OL). In the right configuration the two OsBp moieties are antiparallel and lined away from each other (AP). The shaded rectangle approximates the extent of overlap between OsBp moieties for these three possible configurations. Overlap is extensive with OL, small with PA and negligible with the AP configuration. Statistically the PA configuration is 2-times more abundant compared to the other two. A nanopore is known to sense the direction a strand enters the pore (3′-entry or 5′-entry)^1,2,61^, and it may sense the direction, parallel or antiparallel of a single OsBp with respect to the strand’s direction. This will create four different ways an osmylated nucleic acid can interact with a nanopore, and may yield four different *I*_*r*_*/I*_*o*_ levels (see discussion and Fig. 5a).

HPLC analysis is routinely used for identification, resolution from impurities, stability evaluation, and quality control of pharmaceuticals. RNAs, such as single guide RNA (sgRNA), mRNA, or miRNA, are considered for therapeutic applications, and validated analytical methods to characterize them are being sought^57^. Typically RNA oligos up to the 60-mer can be resolved by ion exchange (IEX)^58^ or ion-pair reversed phase (IP-RP)^59^ chromatography. Both chromatographies were exploited here for analysis of the RNAs used in this study. As HPLC analytical columns (see Experimental Section) we used the ones that in our experience give the best possible resolution, but alternative columns/methods may be put to the test. The 31 nt oligos in this study vary minimally from each other, and their separation and identification by HPLC presents a challenge. Despite method development efforts, complete separation was not achieved. Figure 2a illustrates that 6 out of the 7 oligos appear as a single peak (IP-RP method, top HPLC profile) and 4 out of the 7 oligos do not achieve base line resolution (IEX method, bottom HPLC profile). It should be noted that osmylated RNAs exhibit less resolution by HPLC compared to intact, because the OsBp moiety yields peak broadening. Therefore intact oligos that elute separately may overlap after osmylation.

### OsBp’s topoisomerism

OsBp addition to the pyrimidine C5-C6 double bond occurs from either the top or from the bottom of the pyrimidine ring and yields two topoisomers^42,45,46,60^. Due to the strand directionality these two isomers are detectable by either HPLC or CE (see profiles of osmylated 31 nt oligos in Supplementary Information, part C). Each of the 31 nt osmylated oligos tested here exhibited two peaks with a product ratio not far from unity. It is known that voltage-driven translocation of nucleic acids via nanopores yields different ion current levels depending on the direction of the strand, 3′-entry *vs* 5′-entry^1,2,61^. Since RNA(OsBp) exists in two isomeric forms, it is plausible that a nanopore will sense and discriminate them by exhibiting two different *I*_*r*_*/I*_*o*_ levels. Depending on the strand entry in the pore each isomer may yield two *I*_*r*_*/I*_*o*_ levels for a total of four. In the absence of experiments with immobilized RNAs within the pore^62,63^, it is difficult to assess this proposition. Nevertheless the *I*_*r*_/*I*_*o*_ histogram of T11(ACA) presents four distinct (*I*_*r*_*/I*_*o*_)_max_, supporting the postulate of four *I*_*r*_*/I*_*o*_ levels (Fig. S1, Supplementary Information).

The isomerism of OsBp and its unhindered reactivity towards consecutive bases^42,43^ leads to three diastereomers in an oligo with two adjacent osmylated pyrimidines, as evidenced by CE in the form of 3 product peaks using osmylated T31(CC) and T32(UU) (see Table 1 and Fig. 2b). Figure 2c is a cartoon to illustrate topoisomers from a single or two adjacent pyrimidines. With two adjacent pyrimidines diastereomers result from (i) overlapping OsBp moieties (OL), i.e. lining antiparallel and towards each other, (ii) OsBp moieties lining parallel to each other (PA), and (iii) OsBp moieties lining antiparallel and away from each other (AP). Statistically the distribution of OL:PA:AP is 1:2:1 and the observations with osmylated T31(CC) and T32(UU) support it (Fig. 2b), even though the order of migration appears different for these two materials.

### Nanopore experiments

Motivated by the single Py(OsBp) discrimination in deoxyoligos observed with α-HL^50^, experiments were extended to RNAs, initially using the NanoPatch instrument from Electronic Biosciences (EBS) equipped with a proprietary glass nanopore membrane (GNM) (see Experimental Section). In this platform (NanoPatch/wt α-HL) the discrimination of the nanopore for RNA compared to DNA appeared to be much stronger (Fig. S18 in the Supplementary Information). As described earlier the process of forming the lipid bilayer, achieving single protein pore insertion, introducing the oligo sample without breaking the bilayer and obtaining hour long *i-t* conductance measurements is not, in our experience, a robust and predictable process. Therefore we tested the MinION from ONT, observed translocations using the osmylated RNAs, and present here this work. At the time of this revision there is -still on back order- a 10-fold less expensive flow cell with 126 channels, the Flongle, which requires an adaptor in order to work with the MinION. The Flongle should be more fitting for our application.

The sequences of the tested RNA oligos 22 nt to 100 nt long are listed in Table 1. Their properties, as tested by HPLC, CE, and the MinION, are also summarized in Table 1. HPLC profile of the intact oligo, CE profiles of the osmylation kinetics at low OsBp concentration, the HPLC or CE profile of the osmylated oligo, as well as the histogram of the *I*_*r*_*/I*_*o*_ data determined for each can be found in the corresponding figure in the Supplementary Information. It should be emphasized that the ONT protocol was not followed, no library was created, and the processing enzyme was not included in this study. Hence all the reported translocations are unassisted, driven by the voltage drop, and conducted with a biased voltage in the range −140 mV to −220 mV.

**Figure 3:**
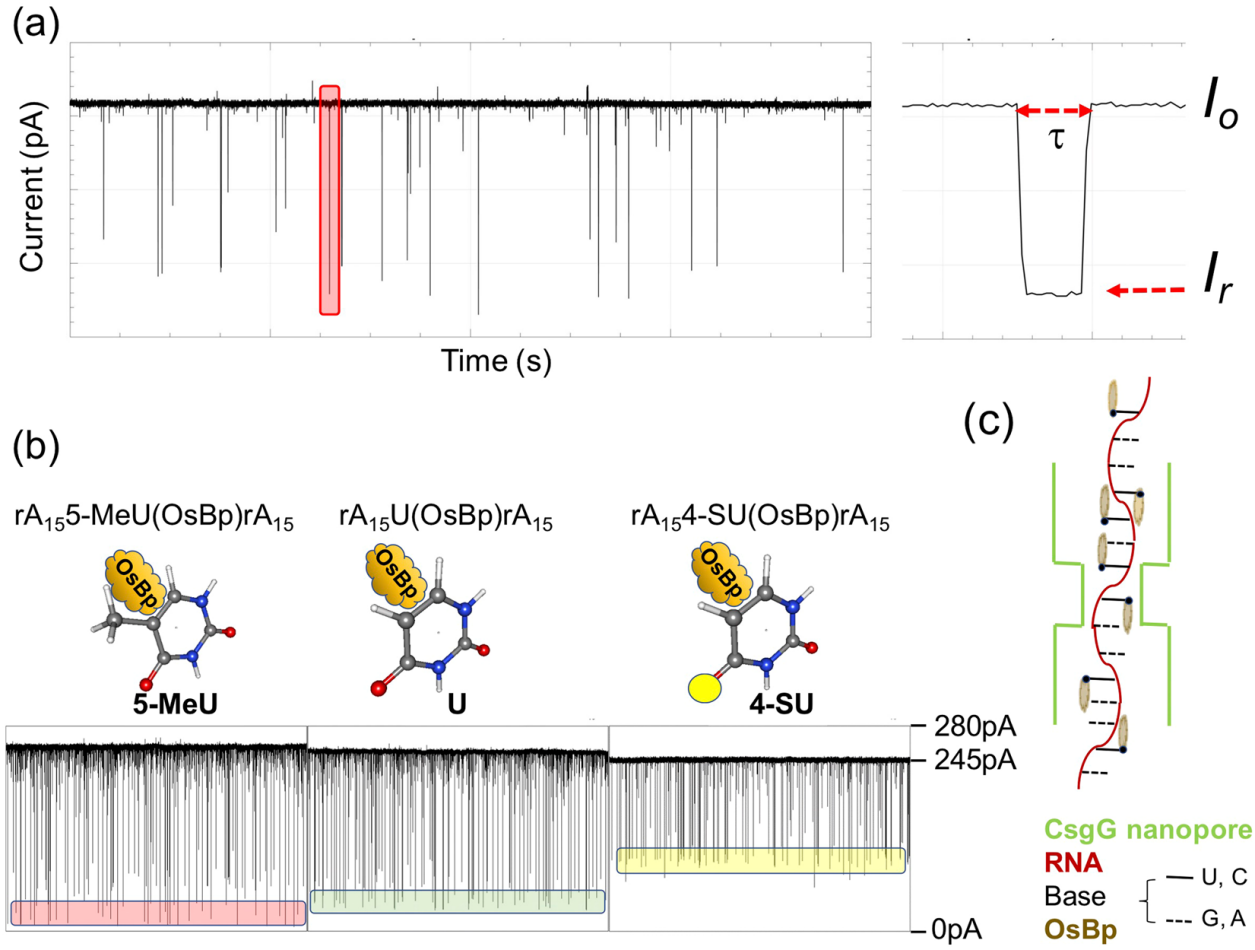
The MinION visibly discriminates three 31 nt oligoadenylates carrying a single osmylated pyrimidine. (**a**) Left: Raw data, 80 s long *i-t* trace at −140 mV and at 34 °C from the unassisted voltage-driven translocation of A_14_GC(OsBp)GA_14_ (T14 in Table 1), using ONT proprietary nanopore/buffer, but no motor enzyme, and not the ONT protocol. Right: Isolated single molecule translocation (pink block on the left) to show open pore ion current, *I*_*o*_, residual ion current of the translocation, *I*_*r*_, and duration, τ. (**b**) Concatenated *i-t* traces from MinION translocations at −180 mV. From left to right: T16(5-MeU), T12(AUA) and T17(4-SU). Top, RNA sequences and below, cartoon of the osmylated pyrimidine base with OsBp at the C5-C6 bond. The presence of a 5-MeU moiety yields the most ion current obstruction (lowest block), less ion current obstruction is observed with U (middle block), and remarkably less ion current obstruction with a 4-SU moiety (highest block); see text for discussion and Table 1 for quantitation of the effect. For data analysis see the corresponding histograms in Figs 5 and S20 in the Supplementary Information. **(c)** Cartoon of the CsGg nanopore with a translocating osmylated RNA. Please note that OsBp is ligned up in a vertical orientation with respect to the ring of the nucleobases and a parallel orientation with respect to the axis of the strand; the orientation of OsBp may be parallel to the 5′-3′ direction of the strand or antiparallel (see Fig. 2c).

Figure 3a represents concatenated *i-t* traces from three experiments each conducted at −180 mV with a A_15_PyA_15_ where Py is 5-MeU, U, and 4-SU. Visual inspection, as seen by the position of the boxes, suggests that the observed translocations with the oligo carrying 5-MeU(OsBp) yield substantially less residual ion current (*I*_*r*_) compared to the translocations with the oligo carrying U(OsBp), and the later yields dramatically less residual ion current compared to the one with the 4-SU(OsBp). The “*I*_*r*_” value at the middle of each box is around 20, 40, and 100 pA for 5-MeU, U, and 4-SU, respectively, spanning a range of 80 pA, somewhat larger than what currently is used for sequencing RNA with the MinION^34^. Figure 3c is a cartoon of the nanopore CsGg used in the MinION and a translocating osmylated nucleic acid strand. The data in Fig. 3b support nanopore-based single Py(OsBp) discrimination in RNAs and this discrimination is made visible using the MinION platform.

### Qualification of the MinION as an analytical tool

To the best of our knowledge, the MinION is exclusively used for enzyme-assisted sequencing. Hence our work regarding voltage-driven RNA characterization is a novel application for this platform. In this context, we evaluated MinION’s suitability as an analytical tool for short RNAs. Three basic questions were addressed: (a) Do all working nanopore channels provide comparable results, (b) Are intact RNA oligos detected by the MinION, and (c) Is the MinION’s pore protein a size-suitable nanopore for osmylated RNA. Briefly the answers to these questions are: (a) and (c) Yes; (b) No for shorter than 31 nt and Yes for longer than 74 nt RNA. The first question was answered by visually inspecting every recorded *i-t* trace from the 512 channels, and by graphing the translocation events (see discussion below) separately for a number of channels. Judging from obtaining superimposable histograms from different channels, channels are comparable. Open pore ion current (*I*_*o*_) from different channels may vary by up to ±15 pA within a single experiment, but data are normalized by reporting the fractional residual ion current (*I*_*r*_*/I*_*o*_) as seen in the histograms in the Supplementary Information. Typically two experiments were conducted with the same oligo using different flow cells, and data are reported from four or more channels for each experiment. It was practically established that 300 *I*_*r*_*/I*_*o*_ values are sufficient to yield a valid/reproducible fingerprint, and 500 to 1400 *I*_*r*_*/I*_*o*_ values are reported per oligo.

**Figure 4:**
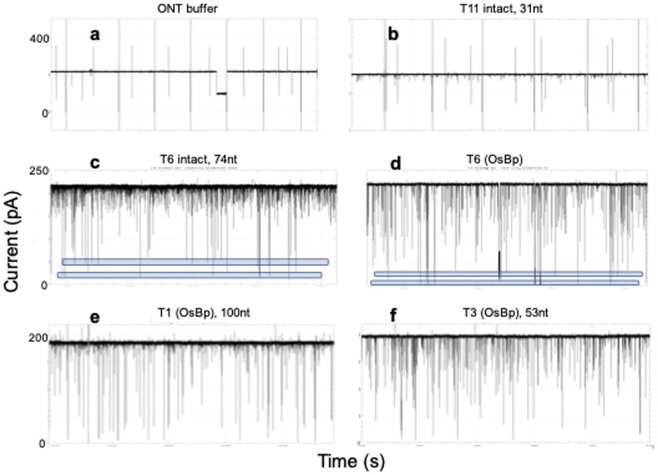
Visual discrimination of osmylated RNA oligos using the MinION. Raw *i-t* MinION recordings from 6 different experiments conducted at 34 °C (not a variable) and at −180 mV. **(a)** ONT buffer as is, 120 s *i-t* trace, observed lines are device-generated and appear more often in the absence of any translocating molecules. **(b)** Intact 31 nt RNA T11 at 4.5 μM, 3x the typical concentration, 100 s *i-t* trace. With the exception of events that exhibit less than 10% current ion obstruction, there are no events that can be attributed to detectable single molecule translocations. **(c)** Intact 74 nt RNA T6, 90 s *i-t* trace, showing a lot of events with high residual current, attributed to bumping events (see text) and a number of events with low residual ion current (two blue blocks). The latter suggests that translocations of intact RNAs as long as 74 nt are recorded by the MinION. **(d)** Same RNA as in c, but osmylated (6 OsBp), 40 s *i-t* trace. The osmylated oligo yields more ion current obstruction compared to the intact, as evidenced by the lower level of the blue blocks compared to the ones with the intact. **(e)** Osmylated T1, 100 nt sgRNA, 50 s *i-t* trace. This RNA exhibits secondary structure^57^, but becomes linearized and traverses the pore after osmylation. This suggests that such RNAs can be characterized via the MinION/OsBp platform. **(f)** Osmylated T3, a 53 nt RNA, 50 s *i-t* trace, at the same (1.5 μM) concentration as T1. The *i-t* trace of 100 nt T1 exhibits remarkably fewer high residual ion current events (*I*_*r*_ > 125 pA) compared to *i-t* trace of 53 nt T3 and 74 nt T6, that are practically oligoadenylates. The observation that oligoadenylates exhibit more bumping events is in agreement with earlier reports using α-HL^2^.

**Figure 5:**
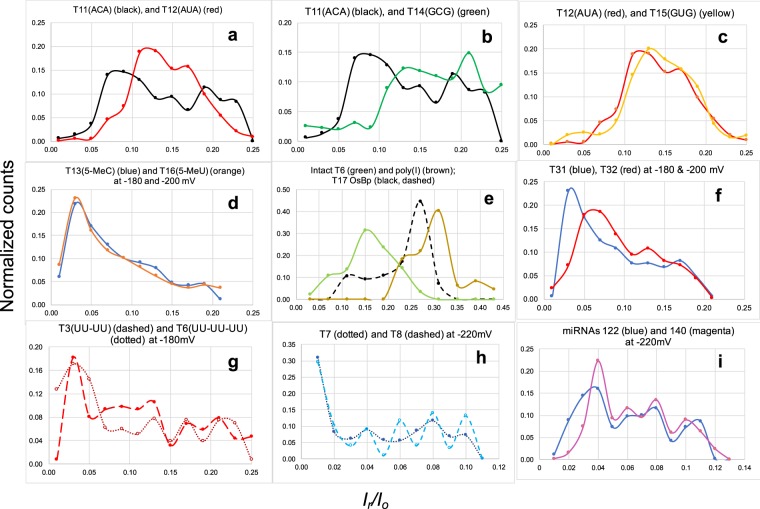
RNA fingerprints: Plots of normalized *I*_*r*_*/I*_*o*_ Histograms from unassisted translocation of osmylated RNA(OsBp) via the MinION. Axes x,y vary among figures in order to match the specific set of RNAs; x-axis, *I*_*r*_*/I*_*o*_ with bin size = 0.01, 0.02 or 0.04; y-axis, normalized counts per bin over total counts. Lowest values (*I*_*r*_*/I*_*o*_)_max_ reported in Table 1. Actual *I*_*r*_*/I*_*o*_ histograms in the corresponding oligo figure in the Supplementary Information. **(a)** T11(ACA) (black) vs. T12(AUA) (red) at −180 mV exhibit distinct profiles. **(b)** T11(ACA) (black) vs. T14(GCG) (green) at −180 mV exhibit distinct profiles. **(c)** T12(AUA) (red) vs. T15(GUG) (yellow) at −180 mV exhibit comparable profiles. **(d)** T13(5-MeC) (blue) vs. T16(5-MeU) (orange) exhibit identical profiles. **(e)** T17(4-SU) (black, dashed) vs. intact T6 (olive green) vs. poly(I) (brown) to show that (*I*_*r*_*/I*_*o*_)_max_ increases in the order T6 intact < osmylated T17 < intact poly(I). **(f)** T31(CC) (blue) vs. T32(UU) (red) exhibit distinct profiles, and comparable to the trends observed in a, but shifted to relatively more ion current obstruction. **(g)** T3(UU-UU) (dashed) vs. T6(UU-UU-UU) (dotted) exhibit comparable profiles, only that counts with the longer T6 are shifted towards more obstruction compared to shorter T3 (see text). **(h)** T7(13Py) (dotted) vs. T8(9Py) (dashed), with similar sequence but different number of Py(OsBp), exhibit comparable profiles, and show the lowest *I*_*r*_*/I*_*o*_ seen in this study, attributed to 5 adjacent Py. **(i)** miRNA122(9/22) (blue) vs. miRNA140(12/22) (magenta) exhibit comparable profiles for translocations at relatively low, but distinctly different at the highest ion current obstruction (see discussion in text). Notably (*I*_*r*_*/I*_*o*_)_max_ = 0.04 for the two miRNAs is in between the (*I*_*r*_*/I*_*o*_)_max_ = 0.03 and 0.06 of the T31(CC) and T32(UU) respectively, consistent with the miRNA sequences containing both C and U bases.

The question whether or not the MinION reports translocations from intact RNA was evaluated by experiments conducted in the absence/presence of intact RNAs. Figure 4a shows the raw *i-t* recording of ONT proprietary buffer. It is noticeable that the open pore current is interrupted by “instrument lines” only, and not by any translocation events. These “instrument lines” traverse the *i-t* trace, and typically exhibit a mirrored image of lines extending vertically up and down. Figure 4b illustrates the raw *i-t* recording from an experiment conducted with an intact 31 nt RNA (T11(ACA)). Figure 4b appears comparable to Fig. 4a, with the exception of a larger number of shallow events that may be attributed to the presence of the RNA. Conducting the experiment at two different concentrations did not show detectable differences; the two concentrations were 1.5 μM RNA which is the typical concentration in this study and a 3-fold higher concentration (shown in Fig. 4b). The similarity of these figures suggests that 31 nt, or shorter, oligoadenylates translocate faster than the instrument’s ability to record them. This observation is in agreement with the reported average 22 μs per adenosine base^2,5^ that estimates τ = 0.7 ms for the 31 nt RNAs in our study, and MinION’s specifications of 3 data points per 1 ms. In contrast, Fig. 4c presents the raw *i-t* recording from an intact 74 nt RNA (T6 in Table 1, estimated τ = 1.6 ms) and illustrates the presence of a large number of translocations, accompanied by a smaller number of “instrument lines” (not shown here due to the choice of y-axis). Some of the events exhibit high residual current *I*_*r*_, and some exhibit low *I*_*r*_. The former are many and considered to be bumping events of the RNA on the pore; the later are fewer and considered to be actual RNA translocations. Within the range of the true translocations events, i.e. below 50 pA in Fig. 4c, one visually identifies the existence of two *I*_*r*_ levels, as shown by the two blue transparent blocks. We attribute these two *I*_*r*_ levels - by extrapolation - to the reported distinct translocations of native nucleic acids via a 3′-end vs. a 5′-end entry into a nanopore^1,2,61^. Notably intact poly(C) and poly(U) translocate via the MinION and exhibit substantially lower *I*_*r*_ levels (*I*_*r*_*/I*_*o*_ ≈ 0.05, 0.10), compared to the one observed with T6 (*I*_*r*_*/I*_*o*_ = 0.15, see below and in Fig. 5e) which is practically an oligo(A). These observations with poly(C) and oligo(A) agree remarkably well with conductance data via α-HL, but differ for poly(U)^1,2^.

Figure 4d represents the raw *i-t* recording of the same molecule discussed above, T6, but osmylated, i.e. carrying six U(OsBp) moieties. A large number of events is observed; some exhibit high residual current *I*_*r*_, attributed to “bumping events”, and some exhibit low *I*_*r*_, attributed to true RNA(OsBp) translocations. The latter are grouped into two *I*_*r*_ levels, as highlighted by the blue transparent blocks. It is noteworthy that the *I*_*r*_ levels considered as true translocations with the osmylated oligo are more ion current obstructing compared to the *I*_*r*_ levels considered as true translocations of the intact. This visible distinction is attributed to the presence of the bulky OsBp tag within the confined space of the nanopore.

To answer the third question regarding suitability of the MinION’s pore for osmylated RNAs, we tested for oligo length and pyrimidine composition to assess (a) whether relatively long oligos, such as the 100 nt sg RNA(OsBp) can traverse the pore, and (b) whether the presence of multiple consecutive Py(OsBp) prohibits translocation. Figure 4e shows a 50 s raw *i-t* trace from an experiment conducted with a fully osmylated 100 nt RNA (see sequence in Table 1), shown to exhibit distinct secondary structure even at 65 °C^57^. As mentioned earlier osmylation linearizes the strand^43^, most likely due to interruption of base-stacking, and this should enable translocation of nucleic acids of any length. Figure 4f shows a 50 s long raw *i-t* trace from an osmylated 54 nt RNA (T3 in Table 1) with a pattern of translocations somewhat different to the one observed with the twice as long T1.

To probe (b) two 32 nt oligos were designed with 9 or 13 pyrimidines, lined in groups with up to 5 adjacent pyrimidines (see T7(13) and T8(9) in Table 1). T7 and T8 share a 3′-end with 5 consecutive pyrimidines, typically found in tRNA sequences. Evidence that adjacent pyrimidines are fully osmylated using Yenos’ protocol, is shown by the agreement between observed and theoretical R(312/272) values; see Table 1 and discussion above. Experiments with these oligos were conducted with a biased voltage at −220 mV, and/or *I*_*o*_ ≈ 270 pA, considered sufficient to induce numerous translocations (see Figs S13 and S14 in the Supplementary Information, and discussion below).

A fourth question that was not fully investigated is whether the MinION may be used for quantitation of osmylated RNAs. The experiments reported here are conducted with a formal oligo concentration about 1.5 μM in the ONT buffer, obtained by dilution in the ONT buffer from a 20 μM RNA(OsBp) stock solution. Considering that the pyrimidine osmylation is practically 100% and that purification leads to practically full recovery with no dilution, it will be of interest to quantify RNA(OsBp) by nanopore and deduce the concentration of a certain RNA in an unknown sample. Limited experiments with dilutions in the range of 3- to 10-fold from the typical concentration illustrate proportionality between concentration and number of actual translocations and supports quantitation. To further probe this issue the software, buffer, and pore temperature, which is currently at 34 °C, of this platform need to be optimized for unassisted translocation of osmylated RNA. Over a period of 10 months a total of 8 different flow cells was used, primarily due to our inexperience with this platform and the novelty of the application. Over 70 experiments were conducted lasting an hour each.

### Acquisition of MinION data, reporting, and results

The software of the MinION, MinKNOW, is currently set up for sequencing experiments, and not for analysis of single molecule translocation data. A recent MinKNOW update includes acquisition of raw *i-t* recordings in *fast-5* file format and direct visualization with MatLab software on a *i-t* plot that includes a units grid, which allows one to precisely determine *I*_*o*_*, I*_*r*_ and τ for any event at an apparent accuracy much better than the device delivers. This feature permits two people, one reading and one reporting, to estimate and report lowest *I*_*r*_ values for every event at ±(1 to 2) pA at a rate of about 700 data per hour. Concatenated *i-t* traces, e.g. the ones reported in Fig. 4, were obtained in house by saving the *fast-5* files in *txt* format readable by open source software QuB (see Experimental Section) and manually “cleaning up” the file to remove instrument lines, and other events that can’t be attributed to single molecule translocations, as will be described next.

Close inspection of the recordings revealed that events, with the exception of the above mentioned “instrument lines” (Fig. 4a,b), can be grouped as follows: (i) Highly noisy events with randomly variable *I*_*r*_, and duration in the range of a few seconds that seem to increase with voltage, oligo concentration, and flow cell use. These events were attributed to noise and ignored. (ii) Relatively shallow and short, τ ≤ 2 ms, events that measure 0.4 < *I*_*r*_*/I*_*o*_ < 0.9, presumably resulting from molecules bumping at the pore; these were also ignored. (iii) Long events with *I*_*r*_*/I*_*o*_ < 0.4 that appear to be the result of multiple translocations, one molecule following the other without reaching open pore current between events; these were also ignored. (iv) Last, but not least, events were observed of low noise with *I*_*r*_*/I*_*o*_ < 0.4 and durations in the range of 2 to 200 milliseconds. We measured 150 to 250 events of group iv (at about 1.5μM oligo concentration) per half an hour of *i-t* recording. We believe that the count of group iv should approach 1000, if one were to count in molecules that translocate as part of group iii, or if the system is optimized so that group iii is suppressed, and replaced by group iv. The observed *I*_*r*_ for group iii is likely not comparable to the corresponding *I*_*r*_ for single molecule translocation due to occupation of the pore by more than one molecule at the time. We attribute group (iv) to single molecule translocations and reported the lowest observed *I*_*r*_ (pA) value for each translocation. Initially we also reported duration (τ) for each translocation (Figs S19 and S20, Supplementary Information), but this type of information, due to the stochastic nature of the process, did not appear to provide added insight for our application. Experiments with 31 nt osmylated T11, T12, T14 and T15 (see Table 1), conducted at both −140 mV and −180 mV (not shown), suggest that mean durations of translocations decrease with increasing voltage, confirming that the events of group iv are true translocations.

Values *I*_*o*_ and *I*_*r*_ were estimated from the *i-t* traces in the Matlab plots, reported together from different experiments and from different channels of the same experiment in Microsoft Excel, *I*_*r*_*/I*_*o*_ calculated from the corresponding *I*_*o*_, and *I*_*r*_*/I*_*o*_ graphed as a histogram with bin = 0.01, 0.02 or 0.04 depending on the oligo; see histograms for each tested RNA in the corresponding Supplementary Information figure. Histograms of two structurally similar RNAs are compared (or fingerprinted) against each other by normalizing the counts of each bin for the total number of translocations as seen in Fig. 5.

### Ir/Io histogram is the fingerprint of an RNA(OsBp)

Each figure in Fig. 5 compares RNAs (Table 1) that differ minimally from each other. In some cases the nanopore senses, and in some other cases it doesn’t sense the structural difference. Figure 5a compares translocation fingerprints of osmylated T11(ACA) and T12(AUA). As a reminder the difference between C and U is that C contains an NH_2_ moiety on C4, whereas U contains an Oxygen (O) on C4, hence the mass unit difference between these two RNAs is 1/10,500, where 1 mass unit is the difference between NH_2_ and O and about 10,500 is the molecular weight of a singly osmylated 31 nt RNA. Figure 5a illustrates that the *I*_*r*_*/I*_*o*_ profile (fingerprint) of T11(ACA) has, perhaps, up to four maxima with (*I*_*r*_*/I*_*o*_)_max_ = 0.08, 0.15, 0.19 and 0.23, whereas the *I*_*r*_*/I*_*o*_ profile of T12(AUA) has two maxima at (*I*_*r*_*/I*_*o*_)_max_ = 0.12 and 0.17. It is presumed that the translocation(s) with the least residual ion current corresponds to a 3′-entry and the one with the higher residual ion current corresponds to 5′-entry, in analogy to the observations with intact nucleic acids^1,61^. The four (*I*_*r*_*/I*_*o*_)_max_ values with T11(ACA) may be attributed to the four different configurations of interaction between a singly osmylated RNA and the nanopore (see Fig. 2c and earlier discussion). These four possible configurations between RNA(OsBp) and nanopore may or may not yield distinct *I*_*r*_*/I*_*o*_ levels, as indicated by the fewer than four (*I*_*r*_*/I*_*o*_)_max_ values observed with most of the 31 nt oligos.

For the purpose of the following discussion the proposition is made that, if a motor enzyme could be engineered to process osmylated RNA one base at a time, then the observed (*I*_*r*_*/I*_*o*_)_max_ values from the unassisted translocations should correspond closely to the ion current levels in the presence of the enzyme. Figure 5a illustrates a 4% difference (from 0.08 to 0.12 pA, i.e. between the two lowest (*I*_*r*_*/I*_*o*_)_max_ values) that, for a typical *I*_*o*_ = 200 pA, translates to 8 pA. A difference of 8 pA suggests that the ACA subsequence is well discriminated from AUA, and that the presence of an enzyme-motor would result to a highly accurate base calling for C(OsBp) vs. U(OsBp). Assuming that the nucleotide ahead of the tagged one (A here), may not contribute much to the observed discrimination, we propose that this platform senses a two nucleotide subsequence, when the first nucleotide is tagged.

Figure 5b compares normalized histograms *I*_*r*_*/I*_*o*_ from the experiments with T11(ACA) and T14(GCG), and illustrates the effect of replacing the adenosine with guanosine. It appears that (*I*_*r*_*/I*_*o*_)_max_ = 0.08 and 0.19 with ACA are now shifted to more residual ion current (*I*_*r*_*/I*_*o*_)_max_ = 0.13 and 0.21 with GCG. This is a major shift and supports the above proposition of sensing a two nucleotide subsequence, when the tagged nucleotide is Cytosine. In contrast to the effect observed with tagged C, tagged U doesn’t result to such large effect, i.e., yielding comparable *I*_*r*_*/I*_*o*_ profiles between T12(AUA) and T15(GUG) (Fig. 5c); hence the corresponding (*I*_*r*_*/I*_*o*_)_max_ = 0.12 and (*I*_*r*_*/I*_*o*_)_max_ = 0.13 may yield a 2 pA (from (0.13–0.12)*200 pA) distinction only. Enhanced discrimination in this case may be achieved in the presence of a second tag to react selectively with Guanosine and leave the other bases intact.

Figure 5d illustrates practically identical fingerprints for osmylated T13(A5-MeCA) and T16(A5-MeUA), pointing out that the presence of a methyl group reduces the ion current so much ((*I*_*r*_*/I*_*o*_)_max_ = 0.03), that the difference in the pyrimidine moiety becomes “silent”. Whether or not replacing A with G adjacent to the 5-Me-pyrimidine will make a difference, remains to be tested. It is noticeable that replacing a canonical pyrimidine with its 5-Me derivative yields 10 pA for C (from (0.08–0.03)*200 pA) and 18 pA for U (from (0.012–0.03)*200 pA) lower maximal residual ion current leading to, perhaps, the most accurate base calling envisioned for 5-Me modifications compared to the unmethylated base. Since the specific nanopore platform does not discriminate 5-MeC from 5-MeU, one could use the higher reactivity of OsBp for 5-MeU over 5-MeC, C, U, and 4-SU (compare part B in Figs S1–S7 in the Supplementary Information), and prepare RNA(OsBp) at low OsBp concentration to preferentially osmylate 5-MeU and leave the other pyrimidines mostly intact. This would be an example where the selectivity of the pyrimidine-specific label assists in discrimination, when the nanopore falls short.

All but one the tested 31 nt RNAs exhibited their lowest (*I*_*r*_*/I*_*o*_)_max_ in the range of 0.03 ≤ (*I*_*r*_*/I*_*o*_)_max_ ≤ 0.13. This range falls below the (*I*_*r*_*/I*_*o*_)_max_ = 0.15 reported for the intact 74 nt T6, and far below the (*I*_*r*_*/I*_*o*_)_max_ = 0.31 observed with poly(I) (Fig. 5e and discussion later). The outlier is the 31 nt T17(4-SU) which exhibits (*I*_*r*_*/I*_*o*_)_max_ = 0.27 (Fig. 5e). The possibility that the observed high (*I*_*r*_*/I*_*o*_)_max_ = 0.27 is the result of shorter oligos, due to phosphodiester cleavage at the 4-SU base, is excluded based on IEX HPLC analysis^57^ of the osmylated T17 that reveals no detectable shorter oligos. The osmylation mechanism in the presence of tertiary nitrogen donor ligands, including 2,2′-bipyridine, is well documented (Fig. 1a)^46^. An explanation based on the reactivity of OsO_4_ towards uracil to form cytosine^64^ is not applicable under our conditions, since there is no ammonia present in the OsBp reagent. Therefore transformation of C = S to C-NH_2_ is impossible. Moreover T11(ACA), which would have been the product of this transformation, translocates with (*I*_*r*_*/I*_*o*_)_max_ = 0.08 and not 0.27 (Table 1). Another explanation based on the reactivity of a catalytic amount of OsO_4_ in the presence of reoxidants, to convert alkenes into cis-vicinal diols is also not applicable^65^ as OsBp contains no reoxidants. In contrast to our observations (Fig. S7 parts B. & C.), diol formation will not exhibit absorbance at 312 nm. CE profiles of the T17 osmylation reaction using the long capillary (see Methods) indicated the formation of two separate products with two topoisomers each (Fig. S7 in the Supplementary Information). All four products were found to be stable at room temperature under extended osmylation conditions, suggesting reaction of OsBp at two locations, perhaps a C4-C5 conjugation in addition to the typical C5-C6 conjugation. Clarity on this issue may await further experimentation.

### Identification of RNA oligos by HPLC vs. nanopore

Due to sequence similarity, discrimination among the seven 31 nt RNAs presents an analytical challenge. Osmylation results in HPLC peak broadening, or formation of two peaks, due to the presence of two topoisomers. Hence HPLC analysis of osmylated oligos will not yield better resolution. A comparison can be made between resolution of the intact oligos by HPLC and discrimination of the osmylated oligos by nanopore. In this context only T17 is easily discriminated from the others by both HPLC and the MinION (compare Fig. 2a with (*I*_*r*_*/I*_*o*_)_max_ data in Table 1). Histograms of osmylated T13 and T16 (Fig. 5d) are identical, histograms of osmylated T12 and T15 are quite similar (Fig. 5c), but histograms of the other three oligos are distinct. The most resolving HPLC method developed by us discriminates between two oligos but yields no baseline resolution among the other four. In addition, resolution by HPLC is typically reduced as a function of oligo length. For example, if the A_15_ tails were to be replaced by A_25_ tails, then HPLC resolution is unlikely. In contrast, a nanopore interrogates the molecule as it passes through, and length is practically a non-issue. Hence nanopore-based characterization of RNA(OsBp) is, in certain cases, superior to intact RNA analysis by HPLC.

### The MinION/OsBp platform senses a 2 nucleotide subsequence

Figure 5f compares normalized *I*_*r*_*/I*_*o*_ histograms of osmylated T31(ACCA) and T32(AUUA) and illustrates their different fingerprints. The observed (*I*_*r*_*/I*_*o*_)_max_ = 0.03 and 0.06 are listed in Table 1. Comparing (*I*_*r*_*/I*_*o*_)_max_ values between T11 (with one C(OsBp)) and T31 (with two adjacent C(OsBp) clearly shows less residual ion current for the latter. The same observation is made between T12 (with one U(OsBp)) and T32 (with two adjacent U(OsBp)). These results support a two-base discrimination in this platform. In addition, T31 exhibits lower (*I*_*r*_*/I*_*o*_)_max_ compared to T32, just as T11 exhibits lower (*I*_*r*_*/I*_*o*_)_max_ compared to T12, indicating internal data consistency. If any, the shorter sequences (T31 and T32) should have produced more, not less, residual ion current, but the length appears to play a smaller role here. Shorter sequences, 22 nt instead of 31 nt, were chosen for T31 and T32 in order to achieve better resolution of the anticipated three diastereomeric products (see CE profile in Fig. 2b). The smaller role of the length with unassisted RNA(OsBp) translocation is also supported by the comparable histograms of 53 nt T3 and 74 nt T6 (see Table 1 for sequences and Fig. 5g for histogram comparison). Figure 5g shows comparable fingerprints for T3 and T6 and (*I*_*r*_*/I*_*o*_)_max_ = 0.03 for both, only that the longer oligo exhibits relatively more counts towards less residual ion current compared to the shorter oligo. This feature can be rationalized on statistical grounds considering that molecules with two U(OsBp) in OL configurations (Fig. 2c) are statistically more abundant with T6 vs. T3. It is noticeable that (*I*_*r*_*/I*_*o*_)_max_ with T3(UU-UU) and T6(UU-UU-UU) measures 0.03, whereas (*I*_*r*_*/I*_*o*_)_max_ with T32(UU) measures 0.06, indicating that the residual ion current inside the pore for unassisted translocations is modulated by a sequence of, at least, 4 + 19 = 23 nucleotides (from UU + UU + 19As). This conclusion is strictly valid for an unassisted translocation that yields a single event, and not applicable to either motor-enzyme assisted translocations or slow translocations that yield sequence information (see later). Figure 5h represents normalized histograms of osmylated T7(13Py) and T8(9Py), illustrates comparable fingerprints and indicates (*I*_*r*_*/I*_*o*_)_max_ = 0.01 for both. The similarity suggests that during unassisted translocation a certain number of OsBp moieties, perhaps 9 out of 32 nt, is responsible for modulating ion current and additional OsBp do not add to obstruction and do not prevent translocation. The extreme case of osmylated poly(C) or poly(U) remains to be tested. In addition to the 100 nt sgRNA (T1), T7 and T8 were specifically designed with five adjacent pyrimidines at the 3′-end to support the expectation that t-RNAs and sgRNAs will translocate via the MinION. Figure 5i compares the normalized histograms of two 22 nt miRNAs^66^ (see more references in the corresponding figures in the Supplementary Information). Figure 5i illustrates similar fingerprints for the two tested miRNAs with a noticeable difference at or below (*I*_*r*_*/I*_*o*_)_max_ = 0.04. To rationalize this difference more miRNAs need to be tested. These two miRNAs could, in principle, be discriminated based on their *I*_*r*_*/I*_*o*_ different histogram profiles, but not within a mixture.

### Characterization/sequencing information in unassisted RNA(OsBp) translocations

The MinION/OsBp platform could find application as an inexpensive quality control assay for selected RNAs, such as described above. It would be even more valuable to use this tool in order to identify and quantify every RNA within an RNA mixture. We are not the first to propose a nanopore-based assay to identify a panel of miRNAs from a blood or urine sample^36,37^, but we believe to be the first to propose a nanopore-based assay to determine purity and impurities in a sgRNA preparation. The first question to ask is whether the MinION/OsBp platform has such potential. On average *i-t* recordings include 500 single molecule translocations per channel per hour, as shown above. Selecting only the ones with low *I*_*r*_ attributed to OL and PA configurations (see topoisomerism discussion and Fig. 2c), one may end up with a mere 20% or 100. Assuming that only 20% of those are longer than, let us say, 10 ms and exhibit inter-event detail, that leaves 20 sequencing-bearing events per channel per hour. If 75% out of the 512 channels in a MinION flow cell are in good working condition, and if the experiment lasts 15 hours, then one expects 384 × 15 × 20 = 115,200 events with sequence-bearing information. Assuming a sample with 200 miRNAs, there should be, on average, a 576-fold representation of each miRNA, more or less depending on their concentration. This “Gedanken experiment” is rather promising, considering that it forgoes improvements in reducing the flow cell temperature from the currently fixed 34 °C, improving sampling rate and/or using a different label. The case in favor of implementing a nanopore-based 100 nt sgRNA purity assay is even more appealing, as impurities should be much less than 200.

**Figure 6:**
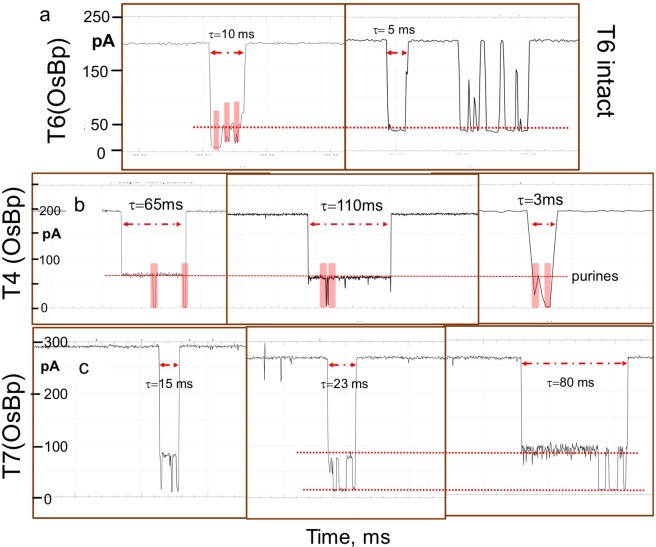
Inter-event detail of single molecule translocations via the MinION to suggest rudimentary “sequencing”, as shown by the different *I*_*r*_ levels, attributed to subsequences of osmylated Py or intact Pu. **(a)** Comparison of T6 translocations at −180 mV between the osmylated (left) and the intact (right) oligo. Observed dips (pink blocks) at lower *I*_*r*_ levels correspond to the three osmylated UU and the red dotted line illustrates the *I*_*r*_ level of the adenosines in between the osmylated UU. *I*_*r*_ levels of UU differ depending on the lining of OsBp moieties with respect to each other (see Fig. 2c and discussion). **(b)** Examples of T4(CC-UU) translocations at −180 mV obtained from different channels, exhibiting different dwell times τ, but with the same number of dips (pink blocks), at low *I*_*r*_ levels, attributed to osmylated Py-Py separated by a higher *I*_*r*_ level attributed to Pu (dotted red line). Three Py-Py *I*_*r*_ levels are expected, but only two are shown here (3 ms event). **(c)** Examples of T7(13Py) obtained at −220 mV from three different channels illustrates the expected three dips of low *I*_*r*_ level to correspond to the three osmylated sequences of consecutive Py and the higher *I*_*r*_ level that corresponds to the sequence of Pu in between.

**Figure 7:**
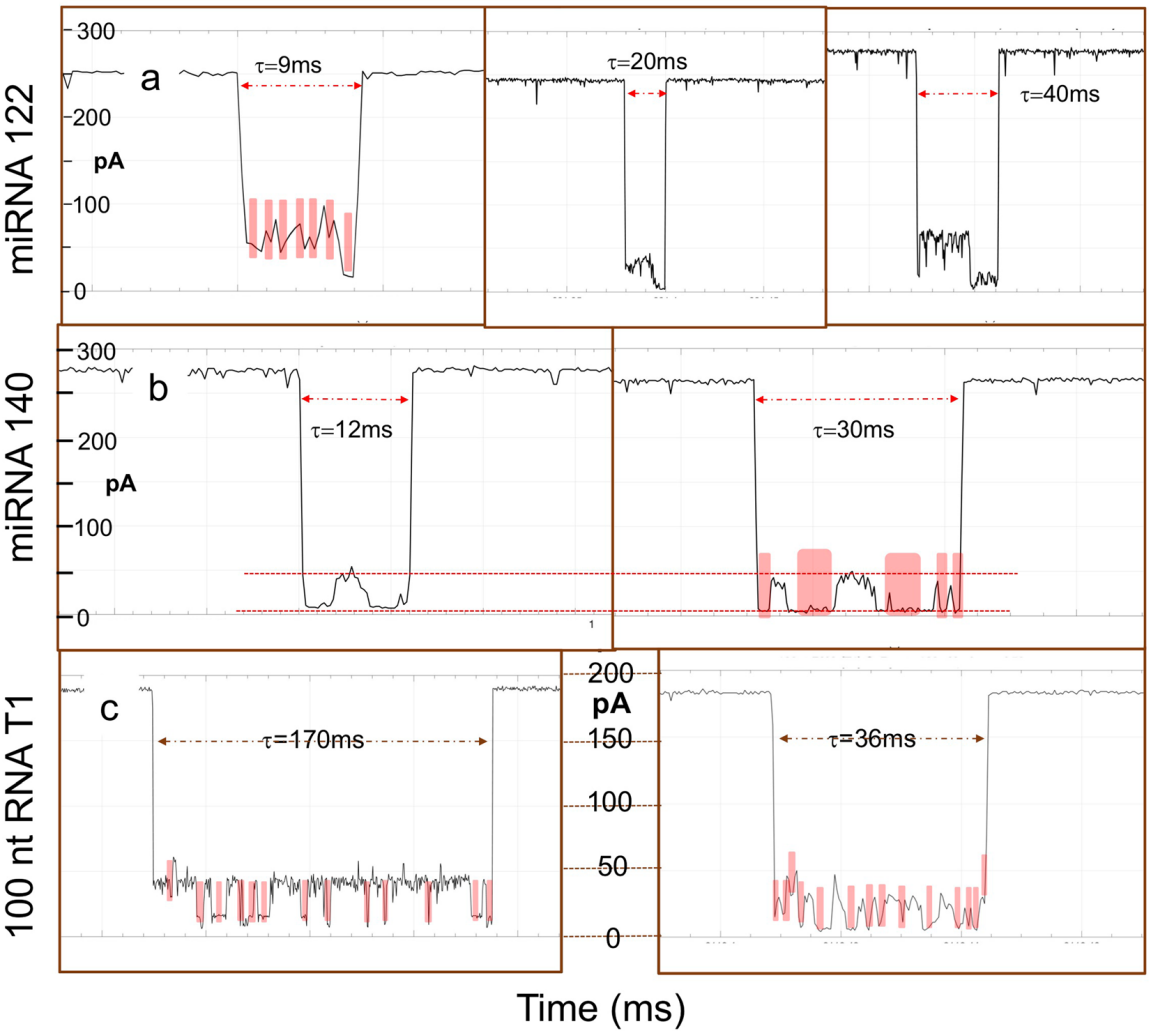
Inter-event detail in single molecule translocations via the MinION to suggest rudimentary “sequencing” of miRNAs and sgRNA, as shown by the different *I*_*r*_ levels within a single molecule translocation, attributed to osmylated Py or Pu subsequences. **(a)** Three examples of miRNA122 translocations taken from two different experiments (left and middle at −200 mV, right at −220 mV). All three are consistent with entry from the 5′-end as evidenced by the higher *I*_*r*_ level that corresponds to single pyrimidines interspersed among purines, and exit from the 3′-end as evidenced by the lower *I*_*r*_ level that corresponds to a sequence of 3 consecutive pyrimidines. The left figure highlights with pink blocks the dips that may be attributed to the six single Py(OsBp), all six at a higher *I*_*r*_ level compared to the *I*_*r*_ level of the last dip that corresponds to the three consecutive Py(OsBp) in the sequence of miRNA122 (5′ **U**GG AG**U** G**U**G A**C**A A**U**G G**U**G **UUU** G 3′). **(b)** Two examples of miRNA140 translocations taken from two different channels of an experiment conducted at −220 mV. Red dotted lines illustrate the higher *I*_*r*_ level attributed to the purines, and the low *I*_*r*_ level attributed to the two subsequences with consecutive Py(OsBp). The translocation with the longer duration (right) appears to match better the sequence of miRNA140 (5′ **C**AG **U**GG **UUU U**A**C CCU** A**U**G G**U**A G 3′), in the form of Py-Pu, as it shows 5 (pink blocks) out of the expected 6 dips. **(c)** Two examples of 100 nt sgRNA, T1, obtained at −180 mV from two different channels, each with 14 dips (pink blocks), consistent with the 14 subsequences of this RNA that contain two or more Py (in bold below). Better resolution is observed with the translocation that exhibits the longest duration of 170 ms. The five consecutive pyrimidines at the 3′end should have produced a much deeper dip, but at −180 mV it is likely that only a truncated version of T1, let us say one missing the last 2 or 3 nucleotides, translocated. It is presumed here that the single Py(OsBp) within a series of Pu yield the observed higher *I*_*r*_ level in between the dips. T1 sequence, 14 regions with 2 or more consecutive Py (in bold): 5′-**UU**A CAG **CC**A CG**U CU**A CAG CAG **UUU U**AG AG**C U**AG AAA UAG CAA G**UU** AAA AUA AGG **CU**A G**UC C**G**U U**A**U C**AA **CUU** GAA AAA GUG GCA **CC**G AG**U C**GG UG**C UUU U**-3′.

We present here a practical approach of how to obtain sequence-bearing translocations, and show such examples in Figs 6 and 7. Translocations of group iv (see above) need to be identified first, the ones with the lowest *I*_*r*_ singled out, and, in turn, the ones with dwell times longer than 10 ms inspected in detail. Lowest *I*_*r*_ for a certain molecule can be approximated using the (*I*_*r*_*/I*_*o*_)_max_ listed in Table 1. Figure 6a compares translocations obtained at −180 mV from osmylated T6 (left) and intact T6 (right). Observed dips (see pink blocks) are attributed to the three adjacent UU. The dip with the lowest *I*_*r*_ level is consistent with OL configuration and the other two with PA configuration (Fig. 2c and caption). The red dotted line represents the *I*_*r*_ level of the adenosines both in the intact T6 and in the osmylated T6. Figure 6b presents examples of osmylated T4(CC-UU) translocations at −180 mV obtained from different channels, exhibiting different dwell times, τ. Each event bears the same number of dips (two pink blocks) consistent with the presence of two sets of Py-Py. The dotted red line represents the *I*_*r*_ level attributed to the oligo(A) subsequence. Notably a 10-fold lower concentration (0.15 μM) used for this experiment led to about a 10-fold decrease in translocations. Figure 6c represents T7, the best molecule in this study to highlight rudimentary sequencing, as it contains three heavily osmylated Py subsequences (4 to 5 OsBp moieties each) separated by Pu sequences. Examples of T7(13Py) translocations obtained at −220 mV from three different channels illustrate the expected three dips at low *I*_*r*_ level that correspond to the three subsequences with consecutive pyrimidines and the higher *I*_*r*_ level that corresponds to the purines (see, dotted red lines).

Figure 7 illustrates the potential of this approach for unassisted characterization of a mixture of miRNAs (Fig. 7a,b) and for a sgRNA (Fig. 7c). Figure 7a presents three examples of miRNA122 translocations from three different channels taken from two different experiments (left and middle at −200 mV, right at −220 mV). All three figures show translocations consistent with entry from the 5′-end, identified by the higher *I*_*r*_ level that corresponds to single Py(OsBp) interspersed among purines, and exit from the 3′-end consistent with low *I*_*r*_ level that corresponds to the three consecutive Py. The left figure highlights the dips (pink blocks) that may be attributed to the six single Py(OsBp) separated by purines, all six at a higher *I*_*r*_ level compared to the *I*_*r*_ level of the last dip that is attributed to the Py-Py-Py at the 3′end. Figure 7b presents two examples of miRNA140 translocations at −220 mV taken from two different channels. Red dotted lines identify two *I*_*r*_ levels: the high *I*_*r*_ level is attributed to Pu, and the low *I*_*r*_ level attributed to the two subsequences each with four consecutive Py(OsBp). The translocation with the longer duration (right) appears to match better the sequence of miRNA140, in the form of Py-Pu. Figure 7c presents two examples of 100 nt sgRNA translocations obtained at −180 mV from two different channels with 14 dips (pink blocks), consistent with the 14 subsequences of this RNA that contain two or more Py(OsBp) (see discussion in the caption). The examples in Figs 6 and 7 suggest that the longer translocations, in the range of 10 to 200 ms, carry inter-event detail reflecting the Py-Pu sequence. Hence optimization of the MinION/OsBp platform towards single molecule characterization is worth pursuing. It is also conceivable that a processing enzyme could be found or bioengineered to bind osmylated RNA or another type of tagged RNA, process it one-base at a time, and sequence it via a two-base discrimination, as seen here.

### Parameters that affect residual ion current in the MinION/OsBp platform

When we proposed to combine nucleic acid labeling with nanopore-based analysis, we expected that the bulkiness of OsBp will limit nanopore selection. The first surprise came by observing translocations of osmylated oligos via α-HL, which has been the prototype protein pore for intact nucleic acids. How is it possible that the increased bulkiness of RNA(OsBp) fits via the same pore size as intact RNA? The second surprise was to observe translocations of T7 and T8 (both with a five consecutive Py(OsBp) subsequence) via the MinION pore protein, albeit at the higher biased voltage of −220 mV. How is it possible to use a platform suitable for intact RNA and detect translocations of overlapping OsBp moieties? The unexpected observations we made with the bulky OsBp fitting pores suitable for intact nucleic acids are not in disconnect with earlier reports^1,2^ and experiments reported here (Fig. 5e) that purines who are about double the size of pyrimidines yield less ion current obstruction compared to pyrimidines. In addition to proposed rationalizations that attribute the observations with intact homopolymer to special helical structures in a confined environment^1,2^, we wish to propose hydration or solvation/desolvation^67^, as another critical parameter, and envision this phenomenon as follows: There are two different sources of water molecules present within the nanopore at all times. One source are the water molecules solvating the salt ions which are responsible for the observed ion current. The other source are the water molecules solvating exposed functional groups of the protein pore amino acids as well as solvating functional groups of the translocating nucleic acid. Within the confined space of the pore less water molecules used for solvation of the pore protein and the translocating nucleic acid directly translates to more water molecules available to transfer salt ions through the pore, or the equivalent, more ion current. Solvation inside a nanopore will not resemble solvation in the bulk solution, but desolvation is costly from a thermodynamic point of view. In the confined space the first, perhaps the second too, solvation shell(s) of any functional group within the pore will remain intact^67^, unless it is replaced by a new component in the system. In this context higher solvation requirements can rationalize the typically lower *I*_*r*_ of ribooligos compared to deoxyoligos, the typically lower *I*_*r*_ of pyrimidines (two functional groups) compared to adenine (one functional group), and the reported here higher *I*_*r*_ level of 4-SU compared to U, consistent with the almost double atomic radius of S vs. O and its resulting easier desolvation. Reduced solvation requirements may also rationalize the apparently lessened effect of OsBp’s size on the ion current reduction as follows: OsBp moiety, with its almost parallel to the strand axis configuration, serves as a “shield”, and by steric hindrance replaces water molecules that otherwise accompany/solvate the nucleobases. The observed extra low residual ion current levels observed in this study, illustrate “touching” proximity between tagged-RNA and nanopore, leading to desolvation and reorganization that enforces superior recognition and discrimination.

## Conclusions

Earlier nanopore studies with intact DNA/RNA exploited an immobilized strand in order to show base-to-base discrimination. Osmylation of nucleic acids adds a bulky tag to all pyrimidines, but does not prevent the modified strand from successfully translocating via protein or solid-state nanopores. Unassisted voltage-driven translocation of osmylated oligos is dramatically slow, and residual ion current is markedly reduced in several nanopore platforms. Here we reported on the translocation properties of osmylated RNA oligos and miRNAs via the MinION device from ONT. We showed that this platform discriminates oligos with structural differences so small that even traditional analytical tools like HPLC may not resolve. For example, conductance measurements visibly discriminate among 31 nt oligoadenylates carrying a single OsBp-tagged pyrimidine. We proposed that this discrimination is the result of slow translocation and close proximity at the narrowest point of the nanopore. The proximity reduces the number of solvating water molecules and enhances interaction/recognition of the chemical components. We demonstrate that histograms of residual ion current from the lowest 25% range serve as the RNA fingerprint, and may be used to confirm/reject proposed sequences in short RNAs, and miRNAs.

The MinION/OsBp platform exhibits substantially reduced residual ion current in the presence of a Py(OsBp)-Py(OsBp), compared to a Py(OsBp)-Pu, indicating a 2 nt sensing, in contrast to the 5 nt sensing observed with intact nucleic acids. Our study illustrates the contribution of the labeling approach for improved recognition in nanopore-based analysis. The MinION and the newer Flongle devices are commercially available, easy to use, relatively inexpensive, and could be further optimized for use with labeled nucleic acids. This proposition is supported by examples of extra slow single molecule translocations bearing multiple ion current levels that mirror the Py-Pu sequence of the tested RNA. RNAs in the range of 20 to 300 nt have important regulatory functions and are currently promoted as biomarkers and/or pharmaceuticals. A simple assay to identify and quantify short RNAs and miRNAs via their Py/Pu sequence may be easy to develop, implement, and commercialize.

## Experimental Section

### Materials and Methods

#### RNAs and other Reagents

Custom-made RNA oligos were purchased from Trilink Biotechnologies or Dharmacon (Horizon Discovery Group); their sequences and the properties of their osmylated derivatives are listed in Table 1. Custom-made deoxyoligos were purchased from Integrated DNA Technologies (IDT). mRNA Cas9 and mRNA EGFP (both CleanCap) were purchased from Trilink Biotechnologies. tRNA(Cys), the E.Coli version by *in-vitro* transcription was purchased from tRNA probes, Texas. Homopolymers poly(C), poly(U) and poly(I) were purchased from Sigma-Aldrich. Purity of oligos was tested by HPLC in-house, typically about 85%, but varies depending on the purification level that was requested from the manufacturer. RNAs were diluted with Ambion Nuclease-free water, not DEPC treated, from Thermo Fisher Scientific typically to 200 or 400 μM stock solutions and stored at −20 °C.

Buffers DNase- and RNAse-free TRIS.HCl 1.0 M pH 8.0 Ultrapure was purchased from Invitrogen and TRIS.HCl 1.0 M pH 7 from Sigma. Triethylamine acetate buffer 2.0 M pH 7 and Sodium hydroxide 1.0 M bioreagent were purchased from Sigma-Aldrich; KCl and NaCl crystalline ACS min 99.0% from Alfa Aesar. 50 mM sodium tetraborate buffer pH 9.3, HPCE quality, from Agilent Technologies. Monomeric protein of the wt α-HL was purchased from List Biologicals, Mountain View, CA. Distilled water from ArrowHead or Alhambra was used for preparation of HPLC mobile phase. A 4% aqueous osmium tetroxide solution (0.1575 M OsO_4_ in ampules at 2 mL each) was purchased from Electron Microscopy Sciences. 2,2′-Bipyridine 99 + % (bipy) was purchased from Acros Organics.

#### Osmylation and purification

OsBp reagent was prepared by preweighing the equivalent of 15.7 mM of 2,2′-bipyridine (bipy) in 18 mL of water in a scintillation vial and adding the full content (2 mL of a 4% OsO4) supplied in an ampule in order to prepare a 20 mL 15.7 mM OsBp stock solution, 1:1 in OsO_4_ and bipy. The concentration of the OsBp stock solution is limited by the solubility of bipy in water and adding OsO_4_ does not increase it, as the complex has a low association constant; OsBp complex represents an approximate 5% of the total, as measured by CE^42^. The low association constant of this complex is also consistent with the dependence of the observed osmylation rate on the square of the nominal concentration [OsBp] (see Table S1 and Figure S17 in the Supplementary Information). Care should be taken that this preparation is conducted in a well ventilated area and that all leftover traces of OsO_4_ are properly discarded. The freshly prepared stock solution is then dispensed in HPLC vials and kept at −20 °C; each vial can be stored at 4 °C and used for a few weeks without loss of potency. It is recommended that every separate stock solution is being validated before first use. To ensure that pseudo-first order kinetics apply we typically use an excess of OsBp at 25-fold or larger compared to the reactive pyrimidine in monomer equivalents. The reactivity of the mononucleotide mirrors the reactivity of the base within an oligo. Manufacturing of osmylated RNAs was conducted typically in 12 mM OsBp, and purification from excess OsBp was done with spin columns (TC-100 FC from TrimGen Corporation) according to the manufacturer’s instructions which takes about 8 min. Close to 100% recovery of RNA is achieved with minor volume/concentration changes, and OsBp reagent is reduced to undetectable amounts after 2-fold purification.

#### HPLC and CE methods

Analyses targeting purity and resolution of oligos of similar sequence were conducted by gradient HPLC; both IEX and IP-RP modes were exploited. Kinetic measurements were primarily conducted by CE, that requires less analysis time compared to gradient HPLC. Analyses were conducted automatically using thermostatted autosamplers. Both CE and HPLC peaks were detected and identified using a diode array detector (DAD) in the UV–vis region 200–450 nm. The electropherograms or chromatograms were recorded at 260, 272 and 312 nm and reported here selectively. Samples were prepared with RNAse free water, but buffers were not. No RNA degradation has been observed in our Laboratory.

For HPLC analysis we used an Agilent 1100/1200 LC HPLC equipped with a binary pump, Diode Array Detector (DAD), a 1290 Infinity Autosampler/Thermostat, and Chemstation software Rev.B.04.01 SP1 for data acquisition and processing. As IEX HPLC column DNAPac PA200 from ThermoFisher Scientific (Dionex) was used in 2 × 250 mm or 4 × 250 mm configurations. The performance of the instrument and the column was qualified using standards every time ahead as well as after analysis of research samples. Two IEX HPLC methods were routinely used and have been validated for purity determination and RNA stability. IEX method at pH 8 is exploiting a 1.5 M NaCl gradient in a 25 mM TRIS.HCl pH 8 buffer, with 30 °C column compartment; typical gradient 85%A-15%B to 45%A-55%B in 12 min where A is 25 mM TRIS.HCl and B is 1.5 M NaCl in A. IEX method at pH 12 is exploiting a 1.5 M NaCl gradient in a 0.01 N NaOH solution (no other buffer needed) with 10 °C column compartment; typical gradient is 100%A-0%B to 5%A-95%B in 16 min where A is 0.01 M NaOH and B is 1.5 M NaCl in A. IEX method pH 12 is validated and recommended for longer RNAs in order to suppress secondary structure that broadens peaks and yields low and misleading resolution^57^. An IP-RP HPLC method was also employed to test RNA resolution using HPLC column DNAPac RP from ThermoFisher Scientific (Dionex) in 2 × 100 mm configuration and flow at 0.35 mL/min. Method IP-RP is exploiting a 25%v/v acetonitrile-water gradient in a 0.1 M TEAA buffer pH 7 at 30 °C or higher column compartment temperature^57^.

CE measurements were conducted with an Agilent G1600 Capillary Electrophoresis (CE) instrument equipped with DAD and Chemstation software Rev.B.04.03(16) for data acquisition and processing; the CE was used in conjunction with a circulating bath to control the autosampler’s temperature. The capillary’s temperature was controlled by the instrument’s software. CE analyses were conducted with an untreated fused silica capillary (50 μm × 40 cm), coined “short” capillary in pH 9.3 50 mM sodium tetraborate buffer using 20 kV or 25 kV. Same buffer was used with an untreated fused silica capillary (50 μm × 104 cm), coined “long” capillary to resolve the topoisomers from the osmylated oligos (Figs 2b and S1–S7, S13, S14, Part C). Both capillaries, short and long, were equipped with an extended light path and were purchased from Agilent Technologies.

#### *Single molecule translocations with wt* α*-HL/EBS GNM membrane (EBS platform)*

A limited number of experiments was conducted with the wt α-HL/EBS membrane (EBS) platform (for examples, see Fig. S18 in the Supplementary Information). Nanopore experiments were conducted with 10 μM synthetic DNA/RNA oligo in 1.0 M KCl, 10 mM TRIS.HCl buffer at pH 8.0 and at 20 ± 1 °C, as described in detail^50,68^, and summarized here. A lipid bilayer was formed across a glass nanopore membrane (GNM) and wt α-HL, purchased from List Biological Laboratories in the monomer form of lyophilized power, was dissolved in water at 1 mg/mL and used in 1–3 μL aliquots to form a single nanopore within a lipid bilayer. The above KCl buffered electrolyte was used to fill the solution reservoir and the GNM capillary. A voltage of 120 mV (trans vs cis) or higher was applied across the GNM between two Ag/AgCl electrodes placed inside and outside of the capillary. The *i-t* traces were filtered typically at 10 kHz and sampled at 50 kHz. Events were extracted using QuB (version 1.5.0.31), and histograms were analyzed by Origin 9.1. Heat plots were plotted using data analysis programs provided by EBS.

#### Single molecule translocations with the CsGg/MinION (ONT platform)

ONT instructions were followed in order to remove air bubbles from the flow cell, add ONT running buffer (RRB or FB), add sample, clean the device after use, and store it. Added samples were either intact oligos or Yenos proprietary osmylated RNA oligos; all the samples were added as is, typically at 1.5 μM concentration in ONT buffer. No library was prepared, and no processing enzyme was added to the sample or buffer, so that all the translocations reported here are unassisted and voltage-driven. Raw data files were acquired in *fast-5* format, visualized directly in MatLab (Mathworks) 2D format. *I*_*r*_*, I*_*o*_, and τ values for each event were visually estimated from the grid at an apparent accuracy better than the one of the device. Microsoft Excel was used to obtain the histograms as shown in the figures (part D) in the Supplementary Information and plot the normalized counts *vs I*_*r*_*/I*_*o*_ bin in Fig. 5. *Fast-5* files were transferred to *txt* format, loaded to QuB open source software (QuB version 1.4.0.1000, the MLab Edition from the Milescu Lab, University of Misssouri and the Sachs Lab at SUNY Buffalo) and concatenated (see Fig. 3b).

## Supplementary information

Nanopore device-based fingerprinting of RNA oligos and microRNAs enhanced with an Osmium tag.

## Data Availability

Some of the data, about 3%, generated during this study are included in this published article (and its Supplementary Information files). All of raw data (each file is about 4GB) may be obtained by request from AK.
